# Heterogeneity in Pancreatic Cancer Fibroblasts—TGFβ as a Master Regulator?

**DOI:** 10.3390/cancers13194984

**Published:** 2021-10-04

**Authors:** Dale M. Watt, Jennifer P. Morton

**Affiliations:** 1 Cancer Research UK Beatson Institute, Glasgow G61 1BD, UK; 2018674w@student.gla.ac.uk; 2 Institute of Cancer Sciences, University of Glasgow, Glasgow G61 1QH, UK

**Keywords:** pancreatic cancer, TGFβ, stroma, fibroblasts, CAFs

## Abstract

**Simple Summary:**

Pancreatic cancer has a very low survival rate and improved treatments are required. In addition to tumour cells, the disease is characterized by a significant non-tumour cell stromal component including immune cells, blood vessels and, particularly, fibroblasts whose normal role is in connective tissue and healing wounds. This stromal environment can affect tumour progression and play a role in response to therapy. Whilst there is relatively good understanding of the deregulated signalling in the tumour cells, less attention has been paid to the stroma until recently. It is now apparent that there is significant variation within and between the stroma in different tumours, in particular, between fibroblast populations. TGFβ is a growth factor that can play a role in cancer cells, but also in shaping the tumour stroma. Here we review recent developments in our understanding of the fibroblast populations within PDAC with a focus on TGFβ signalling.

**Abstract:**

Pancreatic ductal adenocarcinoma is an aggressive disease for which there are very few available therapies. It is notable for its high degree of tumour complexity, with the tumour microenvironment often accounting for the majority of the tumour volume. Until recently, the biology of the stroma was poorly understood, particularly in terms of heterogeneity. Recent research, however, has shed light on the intricacy of signalling within the stroma and particularly the molecular and functional heterogeneity of the cancer associated fibroblasts. In this review, we summarise the recent improvements in our understanding of the different fibroblast populations within PDAC, with a focus on the role TGFβ plays to dictate their formation and function. These studies have highlighted some of the reasons for the failure of trials targeting the tumour stroma, however, there are still considerable gaps in our knowledge, and more work is needed to make effective fibroblast targeting a reality in the clinic.

## 1. Introduction

Over the last 50 years there has been very little improvement in survival of patients diagnosed with pancreatic ductal adenocarcinoma (PDAC) with only 8% of patients in the UK surviving 5 years post diagnosis [1]. Although new chemotherapeutic combinations have been developed over that time [2,3,4], they have failed to provide a cure and shown poor efficacy in terms of improving long-term survival. Surgical intervention remains the best treatment modality, however, this is frequently hindered by anatomical location preventing surgery or resection margin involvement, and survival is limited post-surgery due to recurrence or emergence of metastatic disease. Ultimately, the development of new therapies is required in order to combat the disease, especially with the prediction that PDAC will become the third leading cause of cancer related death by 2030 [5].

With the failure of non-specific chemotherapy, patient stratification remains an attractive option to identify subsets of patients that would respond well to targeted therapies. Constitutively active KRAS is found in 95% of PDAC cases, with mutations in *TP53*, *SMAD4* and *CDKN2A* found in ~50% of cases. However, the publication of multiple human PDAC sequencing experiments has highlighted the abundance of low penetrance gene mutations. These studies also allow for broad clustering of patients into specific cancer subtypes while highlighting commonly dysregulated signalling pathways [6,7,8]. Patient stratification based on either genetic aberrations or transcriptional subtypes may translate to more effective and targeted therapies, such as in a DNA damage repair-deficient subset of patients [9].

Evolving alongside the cancer cells, there is a significant stromal component in pancreatic cancer, consisting of fibroblasts and immune cells and an abundance of extracellular matrix (ECM), together constituting up to 80% of the total PDAC. Although our understanding of the genetics of disease progression has improved, little attention has been paid to this stromal infiltrate until more recently. A large proportion of the stroma is made of cancer-associated fibroblasts (CAFs) which have been proposed to have both a tumour restraining and promoting function [10]. Recently, through single cell RNA sequencing, they have been confirmed to exist as distinct subpopulations rather than a single homogenous population within the tumour [11,12]. These subpopulations have specific localisation and functionality, raising the question of whether specific population targeting through therapy could be possible, although further understanding of these populations is required.

One of the major dysregulated pathways within PDAC is transforming growth factor beta (TGFβ) signalling. The TGFβ signalling family is composed of 33 ligands and in its simplest form involves ligation of a type II receptor which recruits and phosphorylates a type I receptor at the cell surface to propagate intracellular signalling via SMAD molecules. Complexity is imbued by the range of ligands and varied receptor combinations, as well as cell and context specific functions. For instance, TGFβ has been shown to be potently tumour suppressive in disease initiation as illustrated by the frequent mutations to signalling components, TGFβ-receptor-2 (*TGFBR2*), *TGFBR1* and *SMAD4* in human PDAC [6]. This has been confirmed by mouse modelling of the disease, where deletion of the aforementioned genes results in rapid disease progression [13,14]. Yet TGFβ signalling is a driver of epithelial mesenchymal transition (EMT) which is pertinent in driving metastasis in progressed disease. For example, TGFβ secreted by CAFs drives invasive behaviour in PDAC cell lines [15]. TGFβ ligand is abundant within PDAC [14], and, also, has an important role in shaping the tumour stroma. It is a potent suppressor of both the innate and adaptive arms of the immune system [16], as well as acting as an activator of CAFs causing increase production of ECM components as well as increasing contractile functions.

In this review, we will discuss the recent improvements in our understanding of the fibroblast populations within PDAC and the effects of therapeutic targeting, with a focus on the role played by TGFβ signalling.

## 2. TGFβ Signalling Pathway in PDAC

TGFβ signalling has been shown to be a potent tumour suppressor in pancreatic cancer. Disruption of signalling, primarily through *SMAD4* loss, is detected in roughly 50% of PDAC cases, and the tumour suppressive activity of the pathway has been demonstrated in mouse models of PDAC, with deletion of *SMAD4* alongside oncogenic *Kras* accelerating PDAC progression, often via the development of intraductal papillary mucinous neoplasia (IPMN) [14,17,18]. Further, the deletion of *Tgfbr2* also accelerated KRAS-driven carcinogenesis with a median survival of less than 2 months [13], highlighting the importance of TGFβ signalling in epithelial cells. On the other hand, secretion of TGFβ by PDAC stromal cells was found to negatively regulate L1CAM expression and drive a more aggressive phenotype [19]. In contrast with previous studies suggesting a pro-metastatic role for L1CAM [20,21,22], this study showed that L1CAM-low PDAC cells were less differentiated and exhibited enhanced stemness phenotypes, including increased capacity for self-renewal, tumour initiation, migration and invasion and chemoresistance [19]. Interestingly, recent work also found that lowering cholesterol resulted in a switch to a basal phenotype of PDAC, reminiscent of the more aggressive basal/squamous subtype, via a TGFβ1-mediated EMT [23].

TGFβ signalling has been shown to have an important role in the stromal reaction in cancer [24], and specifically in PDAC. For instance, orthotopic injection of Panc1 cells over expressing TGFβ1 has been shown to increase desmoplasia [25]. In vitro, addition of TGFβ to cultured pancreatic stellate cells (PSCs) results in increased expression of alpha smooth muscle actin (αSMA) and increased collagen synthesis [26], but also secretion of matrix metalloproteases indicative of a role in stromal remodelling [27]. Conversely, ECM synthesis by PSCs in response to conditioned medium from PDAC cell lines was abrogated by TGFβ neutralizing antibody [28]. One of the earliest studies of the effects of TGFβ signalling in the pancreas in vivo found that overexpression of TGFβ1 driven by an insulin promoter increased fibroblast infiltration and fibrosis, with the exocrine pancreas almost completely replaced by fibroblasts and ECM deposition [29]. More recently, the dual role of TGFβ signalling in pancreatic cancer was demonstrated in PDAC mouse models with either epithelial or systemic *Tgfbr1* deletion. Epithelial depletion of the type 1 receptor promoted pancreatic tumourigenesis, in line with findings in *Smad4* and *Tgfbr2* deficient models, however, systemic depletion revealed important TGFβ-mediated control of the tumour microenvironment (TME). Tumourigenesis was delayed, production of TGFβ ligand by the stroma was diminished as a result of the disruption of positive feedback caused by receptor depletion, and tumour-associated fibrosis was decreased. In addition, the TME was converted to a less immunosuppressive state, with a decrease in Treg infiltration and significant CD8+ T cell infiltration observed [30]. Further, TGFβ-R2 blockade, in the *p48-Cre; LSL-Kras^G12D^; Cdkn2a^lox/lox^* autochthonous model of PDAC and in orthotopic models, resulted in a reduction in αSMA fibroblasts with a subsequent reduction in collagen deposition, tumour cell differentiation and reduced metastases [31]. Together, these studies demonstrate an important role for TGFβ signalling in the development of the dense stroma associated with PDAC.

In terms of the downstream effects of TGFβ signalling in the stroma, TGFβ treatment of both fibroblasts and CAFs has been shown to lead to increased cell stiffness, and specifically in CAFs, to enhanced cell elongation and spreading, formation of lamellipodia and invasion. Mechanistically, these changes are accompanied by increased Rac, RhoA and ROCK [32]. Biffi et al. (2019) also showed that TGFβ can promote the transformation of fibroblasts to myofibroblastic CAFs in PDAC [33]. This subtype will be discussed in more detail later.

## 3. Fibroblasts within PDAC

In PDAC, a large proportion of the TME is composed of CAFs, which develop alongside the growing tumour and contribute significantly to fibrosis. The ECM of both primary and metastatic sites of PDAC is composed of significant levels of collagens I, III and IV, as well as hyaluronan, with high levels denoting worse prognosis [34]. Stromal cells have been shown to produce more than 90% of the ECM, with collagens comprising the vast majority, and the complexity of this matrix increases as disease progresses [35]. Mouse modelling has revealed that CAFs and malignant cells are intrinsically linked, with a tetracycline inducible oncogenic *Kras* model demonstrating resolution of neoplastic regions, but also of the associated infiltrated stroma, upon ‘switching off’ of oncogenic *Kras* [36]. However, the source of CAFs has yet to be fully resolved due, in part, to the inability to track them in human disease. The general consensus was that they arise from the resident PSCs that become activated by factors released by the neoplastic cells, but recent studies suggest that further investigation is required.

PSCs were initially isolated from the rat pancreas [37] and were shown to become activated in PDAC with increased expression of αSMA and loss of the vitamin A-containing lipid droplets characteristic of quiescent PSCs. [38]. They were shown to be major producers of ECM proteins, including collagens, laminin, fibronectin and hyaluronan, and found to accelerate the growth of subcutaneous tumours when co-injected together with tumour cells in transplant models [26,28,39]. 

It has also been demonstrated, however, that CAFs isolated from human PDAC can arise from mesenchymal stem cells (MSCs). Waghray and colleagues found that a population of these CAFs expressed multiple MSC markers in addition to bona fide CAF markers vimentin, fibroblast activation protein (FAP) and αSMA. These MSC-derived CAFs secreted various cytokines such as IL-6 and IL-8 but exclusively expressed granulocyte macrophage-colony stimulating factor (GM-CSF) differentiating them from other CAFs. The production of GM-CSF from these cells was able to enhance invasion of cultured cancer cells [40].

Further, it has recently been shown that CAF-like cells may be derived from bone marrow (BM)-derived monocytes that infiltrate the tumour. Transplantation of GFP-positive BM into spontaneous mouse models of PDAC highlighted a population of GFP, F4/80 and podoplanin (PDPN) positive cells, a macrophage and pan fibroblast marker, respectively, found even in MSC depleted samples. These cells were shown to be either αSMA positive or negative and able to produce an array of cytokines in culture, including IL-10 and IL-6 [41].

Recently, lineage tracing of Gli1^+^ and Hoxb6^+^ fibroblast within the pancreas highlighted divergent expansion upon oncogenic transformation. Intriguingly, the authors show the existence of both PSCs and fibroblasts (lacking lipid droplets) within the healthy pancreas, with Gli1^+^ fibroblasts found in perivascular regions and Hoxb6^+^ fibroblasts scattered throughout the pancreatic parenchyma [42]. Whereas Hoxb6^+^ fibroblasts failed to expand upon development of neoplasia, the Gli1^+^ fibroblast population was shown to expand and contribute to the αSMA+ fibroblast population during tumourigenesis. However, in terms of αSMA expression, heterogeneity existed across Gli1^+^ lineage cells [42]. Interestingly, there is evidence for reciprocal signalling between the TGFβ and sonic hedgehog (SHH) pathways. In basal cell carcinoma (BCC), Gli1 was found to be induced in response to TGFβ signalling in a Gli2-dependent, but SHH independent manner [43]. Conversely, TGFβ2 was identified as a HH target gene in BCC driven by the essential SHH signalling component, smoothened (SMO) [44]. Crosstalk between the pathways has also been described in human lung fibroblasts. TGFβ1 was shown to regulate expression of hedgehog pathway components, independent of SMO. However, SMO, along with GLI1-dependent transcription, was required for TGFβ-mediated differentiation of normal fibroblasts to a myofibroblast phenotype [45]. The impact of crosstalk between these pathways on CAF function and phenotype in PDAC is, as yet, unknown, but worthy of further investigation.

**Figure 1:**
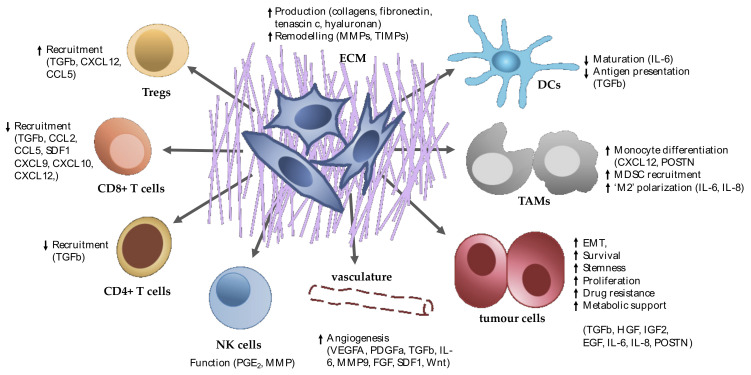
Paracrine CAF signalling influences multiple cell types in the TME.

Together these studies suggest that fibroblast heterogeneity is present in the normal pancreas but is also derived from the infiltration or expansion of cells in pancreatic tumours that can develop CAF-like features and functions. More recently, the field has begun to appreciate that CAFs in PDAC may differ not only in their cell of origin but also in function [46]. These fibroblast subpopulations can respond differently to tumour growth, while also differing in their production of growth factors or cytokines that, in turn, can influence tumourigenesis (Figure 1). Further work, however, is required to ascertain whether CAFs of different phenotypes are derived from distinct progenitors or are educated by their surroundings.

It is important to understand how these heterogeneous populations of CAFs can influence tumour cells and other cells in the TME and to characterize the crosstalk between different cell populations. Indeed, communication between CAFs and tumour cells is highly complex, and not limited simply to one directional paracrine signalling. For example, CAFs and tumour cells can form a reciprocal signalling loop, whereby CAFs educated by the tumour cells can in turn impact oncogenic signalling in the cancer cells. In one study, KRAS^G12D^-transformed cancer cells were shown to regulate the secretome of PSCs [47]. Intriguingly, conditioned media from these tumour cell-educated PSCs could further alter the tumour cell phosphoproteome, beyond the cell-autonomous changes driven by oncogenic KRAS. Most notably, tumour cell autonomous KRAS^G12D^ was sufficient to drive MEK-ERK signalling but not AKT activation, which required secretion of IGF1 and GAS6 by the PSCs [47]. Further, co-culture of CAFs with PDAC cells culminates in enhanced clonogenic growth as well as an increase in cancer cell EMT [48]. Transcriptional and proteomic investigation revealed that matrisome genes are expressed by both pancreatic tumour and stromal cells, and that this is also mediated by tumour-CAF crosstalk in a bromodomain and extraterminal (BET) family protein dependent manner. The BET family of chromatin adaptors, which contain tandem bromodomains allowing interaction with acetylated lysines on target proteins and recruitment of transcriptional complexes to gene regulatory elements, have been implicated in pancreatic tumourigenesis. Disruption of this tumour-CAF crosstalk, using BET inhibition, resulted in reduced tumour growth in vivo [49]. Together these studies point to the importance of the stromal crosstalk in maintaining and driving the signalling required to promote cancer progression. 

As well as stimulating EMT, the fibrotic stroma driven by CAFs has been proposed to impair drug delivery into tumours resulting in highly chemoresistant tumours [50]. Targeting of the stroma has therefore been considered a promising avenue for therapeutic development over the last few years, however, stromal targeting therapies have so far been unsuccessful in the clinic. Recent findings in preclinical models and in human tumour samples have suggested that at least some of the biology attributable to CAFs may restrict tumour progression and have led to a redoubling of efforts to understand CAF biology and functional heterogeneity.

## 4. Inter- and Intra-Tumoural Fibroblast Heterogeneity

Multiple studies have sequenced human PDAC and have exposed a range of cancer specific subtypes, highlighting multiple high and low penetrance mutations across the disease [51]. Until recently, however, sequencing studies focused on analysis of bulk tumour samples, with limited appreciation of the contribution of the tumour-associated fibroblasts. Initial work on dissecting stromal specific signatures by Moffitt et al., utilised bulk RNA data with a ‘virtual microdissection’ to split gene signatures into stromal- and cancer cell-specific gene sets. They highlighted the existence of two stromal subtypes termed ‘normal’ or ‘activated’ by comparison to a stromal defining gene set, as well as a ‘classic’ and ‘basal-like’ subtype derived from the tumour cells. The ‘activated’ stromal subtype was enriched for FAP, WNT family members, and genes related to immune cell recruitment (e.g., CCL13 and CCL18) and ECM remodelling (SPARC, MMP9 and MMP11), whilst the ‘normal’ subtype was enriched for markers associated with PSCs, e.g., αSMA, vimentin and desmin. The stromal subtype was not affected by tumour subtype, with both subtypes observed at similar frequency with either the classical or basal tumour subtype. However, ‘activated’ stroma correlated with reduced survival, regardless of tumour subtype [52]. Studies on RNA extracted from formalin-fixed paraffin-embedded resected PDAC specimens confirmed the classifications of ‘classical’ and ‘basal-like’ from high tumour cellularity samples, however when including all samples, regardless of tumour cellularity, a further three subsets were identified resulting from the impact of the high stromal content and microenvironment-derived signatures. Of these, the ‘immune-classic’ tumours were highly infiltrated by natural killer, T and B cells, but few fibroblasts or inflammatory cells, the ‘stroma-activated’ tumours had higher fibroblast and endothelial cell involvement and low immune cell infiltration, while the ‘desmoplastic’ subtype was characterized by infiltration of fibroblasts, endothelial cells and all immune cell types [53]. Further work, utilising laser capture microdissection to excise stromal and epithelial areas to generate specific transcriptional signatures, again revealed two stromal and two cancer cell subtypes. The stroma ‘ECM-rich’ and ‘Immune-rich’ transcriptional subtypes displayed enrichment for gene sets associated with ECM deposition or immune signalling, respectively, with the tumour epithelium classified as ‘classical’ or ‘basal-like’. Interestingly, in this work the ECM-rich stroma subtype was more likely to be associated with the basal-like tumour subtype, and combined, the ECM-rich stromal and ‘basal-like’ epithelial subtypes correlated with poorer survival [54]. Birnbaum et al. also performed laser capture microdissection of PDAC samples and identified three subtypes of cancer-adjacent stroma (which were named S1–S3) and four cancer cell subtypes (named C1–C4). The stromal subtypes were enriched for gene sets involved in development and cell differentiation (S1), antigen processing and presentation (S2), and phospholipid synthesis and modification of macromolecules (S3). When compared with the Moffitt et al. stromal subtypes, the S1 subtype most closely aligned with the ‘normal’ subtype and was associated with better prognosis, whilst the S2 gene expression signature was enriched in the ‘activated’ subtype [55].

These findings highlighted the inter-tumoural heterogeneity of the PDAC stroma and suggested that a specific stromal signature could influence patient prognosis. They convincingly show that patients can present with different stromal composition within PDAC, with cancer cell subtype not necessarily defining a specific stromal type, although combinations of each may predict worse outcomes. However, there are limitations in analysing CAF-specific functions when analysing bulk stroma, which incorporates CAFs, immune cells and endothelial cells. Therefore, subsequent studies have applied CAF culturing, single cell sequencing (scRNA-seq) and cytometry by time of flight (CyTOF) to investigate, at single cell or population resolution, the fibroblast heterogeneity within PDAC.

RNA sequencing of low passage CAFs derived from human PDAC samples highlighted both inter- and intra-tumoural CAF heterogeneity, with four subtypes defined. From the transcriptional signatures, the authors of this study were able to define specific markers for subtypes A, B and C, (periostin, PDPN and myosin heavy chain 11 (MYH11), respectively), which stained in distinct spatial regions of PDAC [56]. Interestingly, PDPN has been shown to be a pan-fibroblast marker in PDAC in a subsequent study [12]. Here periostin-positive CAFs were located in the centre and at the invasive edge of tumours, whilst PDPN and MYH11 were found only in the centre. Although patients showed intra-tumoural subtype heterogeneity, they could be stratified based upon their dominant subtype from bulk tumour analysis, with subtype A, which also expressed low levels of αSMA, being the most dominantly expressed at both intra- and inter-tumoural level. Subtype D was associated with the poorest prognosis while patients with subtype C enriched tumours showed significantly longer survival than the other groups. In co-culture, subtype A appeared to exhibit a less pro-tumoural phenotype. Interestingly, the authors also found evidence of cancer cell education of the PSCs, with a decrease in the low αSMA-expressing subtype A and an increase in subtypes B and C when exposed long term to tumour cell conditioned medium [56]. In other work, culturing of PSCs in a 2D environment has been reported to cells towards a high αSMA-expressing myofibroblastic phenotype [57], perhaps highlighting the need to consider culture conditions carefully when investigating CAF biology ex vivo. 

Extensive work from the Tuveson lab has uncovered three distinct fibroblast subpopulations within PDAC, termed ‘myCAFs’, ‘iCAFs’ and ‘apCAFs’. Initial work, both in organoid co-cultures and in vivo, documented the existence of myofibroblast-type (myCAF) and inflammatory (iCAF) CAFs. The myCAF population was shown to exist in close proximity to neoplastic cells, expressed high levels of αSMA and TGFβ response genes, and was largely responsible for ECM deposition. In contrast, the inflammatory CAFs (iCAFs), named for their chemokine secreting capability, were found distant from cancer cells, expressed significantly lower levels of αSMA, and instead were characterized by secretion of factors such as Il-6, Il-11, LIF, CXCL1 and CXCL2 [57]. Both subtypes could be derived from PSCs in vitro with appropriate stimulation. Formation of myCAFs could be induced by 2D monolayer culture, but in organoid co-culture, required close-contact with tumour cells. In contrast, only conditioned medium from cancer cells was required for iCAF formation. Interestingly, these phenotypes were dynamic, since alternating culture conditions could mediate subtype switching [57]. Mechanistic work revealed cancer cell-secreted TGFβ and IL-1 as the key drivers of these CAF populations. TGFβ signalling via Smad2/3 was shown to be active in myCAFs, with TGFβ treatment able to drive a myofibroblast phenotype in PSCs in vitro. In contrast, the iCAF phenotype was driven by an IL-1-induced LIF/JAK/STAT pathway. Importantly, TGFβ signalling was shown to inhibit the iCAF phenotype by directly inhibiting IL-1R1 expression, and thus preventing JAK/STAT signalling, suggesting a role for TGFβ is a master regulator of CAF identity in PDAC [33]. TGFβ is secreted in its latent form and its perfusion through the ECM may be limited, perhaps explaining the distinct spatial location of iCAFs distal from the tumour cells. Further single cell sequencing of dissociated human PDAC confirmed the identity of these two CAF populations but also identified a third, antigen presenting, CAF subpopulation, named apCAFs. Interestingly, the S3 stromal signature described by Birnbaum et al. showed significant correlation with this CAF subpopulation [55]. These apCAFs were shown to be MHCII and CD74 positive, and were able to present antigen to T cells in vitro, however, they lack additional co-stimulatory molecules found in professional antigen presenting cells [12]. The distinct advantage these experiments is that the function and location of these CAFs have been defined alongside their distinct transcriptional profile, lending power to the conclusion that these are bona fide CAFs subpopulations. What is less clear is how these CAF populations relate to tumour subtype. One clue came from a study investigating the effects of secreted factors from basal/squamous pancreatic cancer cells on PSCs. This resulted in conversion to CAFs that express high levels of inflammatory cytokines, reminiscent of iCAFs, by a p63 dependent mechanism [58].

Distinct fibroblast populations were also identified in a study in which the authors performed scRNA-seq on normal pancreas, PanIN-bearing pancreas and PDAC from the *Ptf1a^Cre/+^; Kras^LSL-G12D/+^; Ink4a^fl/fl^* (KIC) mouse model of PDAC, before validation in a p53-deficient model. They demonstrated that three populations of fibroblasts were present in normal pancreas and in expanded numbers in early lesions, however, in PDAC this converged to just two fibroblast populations [11]. In the first of these (named FB1), PDAC-associated populations expression of IL-6 and PDGFRα were enriched, while the other population (named FB3) was enriched for the TGFβ-responsive markers αSMA and TAGLN, mirroring the subpopulations definition by Elyada et al., as iCAFs and myCAFs, respectively [11,12]. CD74 and MHCII, markers for the apCAF population, were enriched in, but not exclusive to, the latter population.

Further confirmation of these CAF subtypes was provided by Steele et al., when they analysed a single cell sequencing data set of PDAC [59,60]. However, they go further to suggest that within the three defined overarching subtypes there may exist further distinct populations that are dependent upon other signalling pathways. For instance, SHH signalling was shown to be enriched within a subset of myCAFs compared with iCAFs [60].

Yet another marker that could distinguish CAF populations was identified by the Turley lab. They conducted single cell sequencing of PDPN+ fibroblasts from murine PDAC and observed several transcriptionally distinct CAF populations which converged into two populations driven by IL-1 or TGFβ, reminiscent of iCAFs and myCAFs. The TGFβ-driven population represented ~60% of all CAFs in late-stage tumours, despite being absent in normal pancreas, and was defined by expression of the leucine-rich repeat containing 15 (LRRC15) protein. This population was also observed in human tumours, with clinical trial data showing that the LRRC15+ CAF signature was associated with poor response to immunotherapy, suggesting an immunosuppressive role for these cells [61].

Other CAF markers which imply CAF plasticity have been identified. For example, Fujiwara et al. found that the putative mesenchymal stem cell marker, CD271, was elevated in the stroma of PDAC compared to normal pancreas, predominantly at the tumour periphery. However, high stromal CD271 expression was associated with improved prognosis in pancreatic cancer patients. In PSC-tumour cell co-culture experiments, CD271 expression in PSCs was increased initially, but decreased upon prolonged co-culture, suggesting that expression decreases after prolonged exposure to tumour cells [62]. A more recent study also found that CD271 expression was elevated in peripheral CAFs compared with those in close proximity to tumour cells. Intriguingly, however, in patients receiving neo-adjuvant FOLFIRINOX (folinic acid, 5-fluorouracil, irinotecan and oxaliplatin), regressive stroma became evident and CAFs within these regions now exhibited higher CD271 expression [63]. These data raise the possibility that chemotherapy can mediate a switch in CAF subtype. However, the functional relevance of CD271 expression is yet to be explored. 

Recently, the transcription factor, paired-related homeobox 1 (PRRX1), has also been suggested to play a role in CAF plasticity. It was reported to be highly expressed in CAFs in both mouse and human PDAC, with stromal PRRX1 expression correlating with the basal/squamous PDAC subtype. In mouse models, fibroblast-specific *Prrx1* deletion led to more differentiated, less metastatic tumours, alongside changes to the tumour ECM, and this phenotype was attributed to release of hepatocyte growth factor (HGF) driving EMT in tumour cells [64]. This was reminiscent of, yet in contrast to previous findings describing isoform-specific roles of PRRX1a and 1b in pancreatic cancer cells, with PRRX1b promoting HGF-mediated invasion, tumour dedifferentiation and EMT in the primary tumour, and PRRX1a driving differentiation and mesenchymal-epithelial transition at distant sites [65]. One open and interesting question is whether PRRX1 in CAFs could play a role at distant sites. Intriguingly, *Prrx1*-deficient CAFs recapitulated many of the features of myCAFs, but unlike myCAFs, were fixed in their activated state when cultured in Matrigel, suggesting that PRRX1 may be key for mediating CAF plasticity.

Recently, the Jørgensen lab described an alternative CAF classification based on expression of CD105, a co-receptor for TGFβ. Using mass cytometry to analyse the stroma in pancreatic tumours arising in autochthonous mouse models they identified two functionally distinct pancreatic fibroblast lineages which were also present in normal pancreas as well as in human PDAC and normal healthy pancreas. The CD105+ fibroblasts were more abundant in tumours, however, CD105- CAFs were more proliferative within tumours, and a minority of tumours showed an abundance of CD105- CAFs [66]. Of the previously described CAF markers, most showed variable expression in both CD105+ and CD105- CAFs indicating the presence of both myCAFs and iCAFs in each population although the CD105- population was enriched for apCAF markers. Functionally, TGFβ signalling was enriched in CD105+ CAFs whilst TNF-α, NF-κB, IL6, JAK2 and STING1 signalling were differentially enriched in the CD105- population. Interestingly, and in contrast to the iCAF/myCAF classification, CD105+/ CD105- CAFs remained ‘locked’ in their initial state during long term mono-culture and, also, in response to treatment with TGFβ1 or incubation with tumour cell-conditioned medium or in tumour cell co-culture. In cultures of normal pancreatic fibroblasts (PaFs), the ratio of CD105+ and CD105- fibroblasts was also maintained in response to most treatments, with the exception of TGFβ1, which increased the proportion of CD105+ PaFs, and TNFα and IFNγ, which increased the proportion of CD105- PaFs. IFNγ also caused an increase in apCAF markers in both CD105+ and CD105- fibroblasts, which could be inhibited, again in both populations, by TGFβ1 [66]. 

In co-transplant experiments in mice, CD105+ PaFs were permissive for tumour growth in that they did not significantly influence tumour volume. However, CD105- PaFs were revealed to be highly tumour-suppressive—co-injection of this population markedly constrained tumour growth and led to improved survival, an effect recapitulated even when a 1:1 mixed population of fibroblasts were co-injected. CD105- co-injected tumours showed increased infiltration of dendritic cells (DCs) and CD8 T cells, and pathway analysis revealed an enrichment for pathways involved in tumour-suppressive immune responses including DC maturation and T cell activation, suggesting this tumour-restrictive effect is dependent on adaptive immunity. Indeed, the tumour suppressive effect was lost in immunodeficient mice and in mice lacking conventional type 1 dendritic cells (cDC1s). Although no signalling capacity has been reported downstream of CD105, it has been shown to alter the affinity of ligands for the TGFβ receptor complex and deletion of *Eng*, the gene encoding CD105, from CD105+ CAFs did reduce transcriptional responses to TGFβ1 treatment. Interestingly, however, any function attributable to CD105 did not play a role in tumour suppression, since *Eng* deletion from CD105+ cells was not sufficient to affect tumour growth [66].

**Figure 2:**
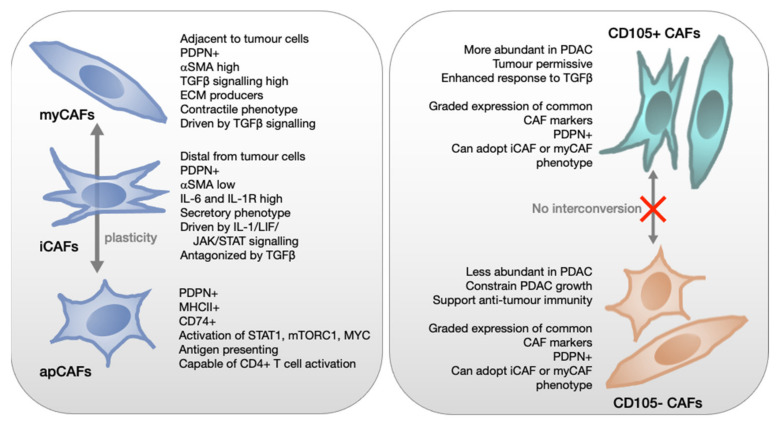
CAF subtypes.

Collectively, these papers highlight inter- and intra-tumoural heterogeneity through multiple techniques (Figure 2).

It is worth noting the caveats of these single cell systems, which involve the dissociation of the tumour with the potential of altering the cell transcriptome. However, combining these data with functional data and in situ validation in tumour tissue has allowed verification of the findings of the single cell experiments. Although our understanding continues to improve, a consensus is still to be met upon fibroblast subpopulations definitions. Across studies, subtypes have varied in definition, with different IHC markers defining different subpopulations. It is key, not only to pinpoint bona fide markers for these subsets, but also to better understand the function of these sub-populations and the effects of their manipulation in complex tumours in order to enable the stratification of patients and understand how variations in CAF composition of patient tumours might represent therapeutic vulnerabilities.

## 5. Genetics of PDAC Influence CAF Education

Adding a further layer of complexity, the genetics of PDAC may directly impact the TME with, for instance, the loss of *SMAD4* resulting in a stiffer fibrosis which correlates with a poorer survival [67]. Work in *Tgfbr2* deficient mice highlighted a similar phenotype with a stiffer ECM in comparison to the gold standard KPC (*Pdx1-Cre*; LSL-*Kras*^G12D/+^; LSL-*Trp53*^R172H/+^) model [67], and this was found to be mediated by JAK-STAT3 signalling. In addition, the authors of this study described a feed-forward loop, by which the TME amplifies JAK–STAT signalling and a β1-integrin–FAK–ROCK mechanosignalling cascade to promote tumour aggressiveness [67]. Interestingly, in their CAF depletion study, Ozdemir et al. utilised *Tgfbr2* deficient mice to show that myofibroblast depletion led to more aggressive tumours and reduced survival, although they obtained similar results in the KPC model [68].

Recurrent mutations in the SLIT/ROBO pathway have been described in human PDAC [69] and may also impact on TGFβ signalling and the TME. For example, loss of epithelial ROBO2 expression and coincident upregulation of ROBO1 in the stroma has been reported in PDAC. In mixed cell cultures from the pancreata of mice with pancreatic epithelium-specific *Robo2* deletion, expansion of ROBO1+ activated myofibroblasts was observed along with induction of TGFβ signalling. In vivo, the *Robo2*-deficient mice exhibited TGFβ-dependent increased fibroblast activation, fibrosis and immune signalling markers in response to experimental pancreatitis, while in PDAC patients, low ROBO2 expression high ROBO1 expression were associated with poorer survival. Thus, perturbations in ROBO1/2 signalling could indicate a patient subgroup who could respond to TGFβ or stromal targeting agents [70].

Even different mutations within the same gene may affect CAF biology. CAFs from p53^R172H^ mutant KPC tumours were shown to create a more collagen dense environment in comparison to those isolated from p53-deficient KPflC (*Pdx1-Cre*; LSL-*Kras*^G12D/+^; Trp53^fl/+^) tumours [71]. The gain-of-function p53^R172H^ tumour cell-educated CAFs created a stiffer matrix that could enhance the invasion of the less metastatic p53-deficient cancer cells, potentially via secretion of perlecan, which was elevated in mutant p53-educated CAFs, as well as in the stroma of KPC vs. KPflC PDACs. Interestingly, given its role in driving the iCAF phenotype, TNFα was able to enhance perlecan expression by the KPflC educated CAFs [33,72]. Gain-of-function p53 mutations have also been shown to correlate with increased ECM deposition and the exclusion of cytotoxic CD8^+^ T cells in mouse and human PDAC [73].

Epigenetic changes may also influence CAF plasticity. For example, matrisomal gene expression was shown to be dependent on BET family proteins mediating crosstalk between tumour cells and CAFs, with a small molecule inhibitor able to alter matrisome gene expression and inhibit tumour growth in mouse models [49]. Xiao et al. also showed that tumour cells can influence CAFs through epigenetic changes. In co-culture experiments they found that direct contact with PDAC cells led to methylation of SOCS1 in CAFs, via the DNA methylation enzyme DNMT1. SOCS-1 methylation and consequent downregulation resulted in enhanced STAT3-induced IGF-1 expression by the CAFs and promotion of cancer cell proliferation and survival [74]. Further epigenetic analysis of CAFs from human PDAC revealed extensive loss of cytosine methylation with multiple genes encoding inflammatory secreted factors, including CXCR4, showing differential hypomethylation resulting in overexpression. Inhibition of CXCR4 could reduce CAF-mediate invasiveness of cancer cells in co-culture. Mechanistically, lactate secreted by PDAC cells was shown to lead to production of alpha-ketoglutarate in mesenchymal stem cells, which in turn activated the ten-eleven translocation (TET) demethylase enzyme leading to decreased cytosine methylation and elevated hydroxymethylation during conversion to CAFs [75]. Interestingly, the basal/squamous PDAC subtype has been associated with loss of hydroxymethylation via reduced expression of the TET2 5-methylcytosine hydroxylase. Importantly, however, in this study the loss of 5-hydroxymethylcytosine was restricted to pancreatic epithelial cells, with levels remaining high in stromal cells [76], in keeping with the previous findings. Intriguingly, the authors did show that, at least in tumour cells, loss of SMAD4 expression correlated with reduced hydroxymethylation, implicating TGFβ signalling as an additional regulator of hydroxymethylation.

It is not surprising to note that the genetic aberrations within PDAC, which have the capacity of altering the wider TME through direct and long-range signalling, can influence the CAFs. It does, however, add complexity to our understanding of CAF subtyping with the predominance of work focussing on the KPC mouse model, reflecting the frequent gain of function p53 subtype of human patients. It will be interesting to discover whether the current CAF subtyping definitions are similar in other genetic models of PDAC, and whether the subtype proportions change with differing genetic education. Examination of late-stage tumours in the KIC, KPC and *Pdx1^Cre^; Kras^LSL-G12D/+^; Trp53^fl/fl^* mouse models of PDAC has highlighted a similar clustering of CAFs [11], suggesting that these genetic permutations do not greatly affect the overarching sub-classification of CAFs at least at transcriptional level. In support of this, similar CAF subsets have been observed across different cancer types with varying genetics, including breast, ovarian, head and neck and lung cancer [77,78]. Conversely, tumour cells can elicit diverse effects on CAF phenotype, irrespective of genetic aberrations. For example, the Trumpp lab described two PDAC subgroups, based on DNA methylation and expression of an IFN-linked transcriptional program, with methylation low/IFN high tumours exhibiting a more aggressive phenotype. These subtypes were linked not to tumour genetics but to cell-of-origin, with ductal lineage tumours exhibiting the IFN high signature. Investigation of the impact of tumour cell subtype on CAF programming revealed differential gene expression in PSCs cultured in medium conditioned by tumour cells of these subgroups, with IFN high subtype tumour cells driving expression of genes associated with inflammatory programs, reminiscent of iCAFs [79]. However, in most of these studies, it should be noted that the functionality of CAF subtypes across different models has not yet been examined.

## 6. Therapeutic Intervention

With the general failure of frontline therapies in the treatment of PDAC, extensive research has been conducted to identify new therapeutic avenues. Stratified therapy appears an exciting option due to the ability to cluster patient subsets based on genetic permutations, which may expose new vulnerabilities. However, as our understanding of fibroblasts heterogeneity increases, this field may provide new targets to influence ‘good’ or ‘bad’ fibroblasts or to reprogram the TME.

Early studies in PDAC suggested that the fibrotic stroma could restrict chemotherapeutic penetrance, and therefore proposed therapeutic targeting of stromal elements in combination with chemotherapy. In 2009, work by Olive and colleagues in PDAC mouse models found that targeting SHH signalling, a known driver of desmoplasia in pancreatic cancer [80], could deplete the tumour stroma, improve tumour vascularity and enhance delivery of standard-of-care gemcitabine into tumours, resulting in increased survival [81]. The suggestion that the stroma could limit drug penetration in PDAC was supported by work in mouse models targeting the ECM component, hyaluronan, which resulted in normalization of interstitial fluid pressures and microvasculature, and improved efficacy of gemcitabine [82]. Attempts to translate these concepts to the clinic, however, were disappointing [83,84].

Subsequent preclinical studies attempted to understand the reasons for these failures. Genetic models assessing the effects of pancreatic epithelial *Shh* deletion, chronic pathway inhibition or depletion of αSMA fibroblasts resulted in more aggressive, poorly differentiated tumours and reduced survival in KPC mice [68,85,86]. Interestingly ‘basal-like’ tumours, which are more undifferentiated, have been shown to have a worse prognosis but the presence of stromal signatures has been shown to improve their survival [53]. Work by Steele et al., showed that SHH signalling was enriched in a subset of myCAFs [60], suggesting that pathway inhibition is predominantly affecting this population, and demonstrated a shift in proportions of CAF subtypes to favour iCAFs upon SHH pathway inhibition [60]. The iCAFs have been shown to release cytokines such as IL-6 [33], which is linked to cachexia and generally a worse prognosis in PDAC [87], and this may explain the failure of SHH targeting in the clinic. Insight on this could be gained from Huang et al., who showed that inhibiting stromal TGFβ-R2, in PDAC in mice lacking TGFβ-R2 in the epithelial cells, resulted in reduced levels of IL-6 and therapeutic benefit. They found that IL-6 high fibroblasts had the highest expression of *Tgfbr2*, and although canonical TGFβ signalling is thought to antagonize iCAFs, TGFβ stimulation of CAFs led to upregulation of IL-6 and LIF via non-canonical signalling through JunD [88]. 

Recent studies have sought to target CAF function, rather than ablating whole populations. For example, perlecan deletion from KPC tumour-educated CAFs promoted sensitivity to gemcitabine–abraxane combination therapy in a co-transplant orthotopic model [71]. In contrast, deletion of collagen I specifically from αSMA^+^ fibroblasts, accelerated pancreatic tumourigenesis in a dual recombinase mouse model of PDAC. Interestingly the lack of collagen I in this situation did not affect the proportions of fibroblast subpopulations, as determined by single cell RNA sequencing, but did result in poorly differentiated, immunosuppressed tumours. Although αSMA is expressed across most CAF subtypes, the myCAFs exhibit the highest expression and are also the major producers of ECM. Interestingly, collagen I deletion from fibroblast specific protein 1 positive fibroblasts had no effect on survival [89]. This study does, therefore, suggest that at least a subset of myCAFs may be tumour suppressive. However, in contrast to these findings, Losartan, an angiotensin inhibitor with anti-fibrotic effects, previously shown to inhibit collagen I production by CAFs and reduce stromal collagen in a pancreatic cancer mouse model [90] was able to reduce both stromal collagen and hyaluronan, potentially via inhibition of TGFβ1, and allow enhanced drug delivery [91]. Indeed, a phase II trial of Losartan in combination with FOLFIRINOX showed promise in the locally advanced PDAC setting [92]. Therefore, further, clarity is required on the role of collagen production by different CAF populations, and gene deletion in the iCAF subpopulation specifically may enhance this understanding.

Enhanced drug efficacy in tumour cells has been a goal of many studies investigating stromal manipulation in PDAC. However, conversely, tumour cell-targeted therapies may also have consequences in the TME and on the CAFs. In general, CAFs appear to be resistant to gemcitabine in terms of survival, indeed, they have been reported to sequester active gemcitabine metabolites, thus protecting tumour cells [93]. Their response to treatment can, however, significantly influence the surrounding cancer cells. For example, gemcitabine has been shown to promote the release of extracellular vesicles from CAFs, which led to increased proliferation and drug resistance in the pancreatic tumour cells via increased snail expression [94]. CAF-derived IGF-1 and IGF-2, as well as SDF1, have also been implicated in resistance to standard of care chemotherapies in pancreatic cancer [95]. Thus, it is worth noting that increasing drug penetration alone is unlikely to lead to significant advances in terms of efficacy. The availability of active drug metabolites and the inhibition of stromal signals able to confer chemo-resistance are likely to be more important than drug delivery.

Chemotherapy can also indirectly affect the output of CAFs as a consequence of deregulated stromal-tumour crosstalk. For example, Abraxane treatment can drive CXCL10 expression by PDAC cells, resulting in diminished CAF secretion of IL-6 and reduced cancer cell migration and invasion [96]. Long-term therapy with gemcitabine in PDAC mouse models was shown to drive increased antigen presentation and immune checkpoint expression as well as elevated TGFβ signalling and cytokine and chemokine secretion. These factors resulted in profound changes in the composition of the tumour stroma, rendered the CAFs resistant to gemcitabine and drove further TGFβ1 release [97]. Treatment with a combination of gemcitabine, the TGFβ-R1 inhibitor, galunisertib, and immune checkpoint blockade resulted in restoration of anti-tumour immunity and extended survival in KPC mice [97]. Of note, an interesting observation of increased CD105 expression was seen in long-term KPC treated mice tumours, but not in cancer cell lines, which could be of particular interest with Hutton et al., showing a divergent function of CD105^+^ CAFs vs. CD105^-^ CAFs [66].

Of course, care needs to be taken when employing TGFβ-targeting strategies. In the tumour cells, TGFβ signalling has clear tumour-suppressive effects, and TGFβ inhibition alone had been shown to exacerbate disease in mice in both early and late intervention studies [98]. Nevertheless, the TGFβ receptor inhibitor, galunisertib, combined with gemcitabine, improved overall survival in unresectable patients compared with gemcitabine [99], although this agent has since been discontinued. Inhibition of TGFβ-R2 has also been shown to reduce collagen deposition and immune suppression in orthotopic and autochthonous models of PDAC [31]. Further, models with cancer cells bearing *Tgfbr2* mutations showed an increased immune infiltration when treated with a TGFβ-R2 inhibitor, thereby depleting TGFβ signalling in stromal cells only [88]. In contrast, Zhang and colleagues found that Tregs represent a significant source of TGFβ ligands in PDAC and observed that Treg depletion resulted in fibroblast reprogramming towards an inflammatory phenotype, with myeloid cell recruitment, immune suppression and accelerated tumourigenesis [100].

Cytokine education of CAFs has also been shown to impact on their function and may yet provide a therapeutic opportunity. For instance, IL-17 deletion in the KPC mouse altered the TME compared to controls, with increased IL-17F in the serum and higher expression of its receptor specifically in the CAFs in IL-17 deficient tumours. The CAFs from IL-17 deficient tumours exhibited significant gene expression changes including elevated TGFβ signalling components. Fibrosis was enhanced but crucially, the stroma was of a less compact and more immune-permissive nature resulting in increased T cell infiltration and suggesting the use of IL-17 inhibitors as part of combinatorial strategies [101].

These studies all point to an important role for TGFβ signalling in the wider PDAC TME, but further work is needed to understand how this can be translated safely for therapeutic benefit. The pancreatic cancer stroma is generally believed to be an immunosuppressive environment, and this is likely mediated in part by the secretion of immunosuppressive factors such as TGFβ since TGFβ can regulate the function of many different immune cell types [102]. TGFβ signalling in the tumour stroma has been implicated in resistance to immunotherapy and studies in mouse models of other tumour types have shown that immunotherapy can synergise with TGFβ blockade [103,104]. In pancreatic cancer, predominance of the TGFβ-driven, LRRC15+ CAF population correlated with poor outcome in response to immune checkpoint blockade [61], whilst a phase I trial of M7824, a bifunctional fusion protein designed to simultaneously target TGFβ signalling and PD-L1, showed promise in a small number of pancreatic cancer patients [105]. A role for TGFβ in suppressing cytotoxic T cell function in PDAC has been described, with TGFβ inhibition leading to increased PD-L1 expression and abrogation of anti-tumour immunity [30,106]. Thus, the combination of TGFβ inhibition with immune checkpoint blockade has been investigated by several groups. Studies in mice have shown that inhibition of TGFβ with checkpoint inhibition can improve survival, with reduced fibrosis leading to increased infiltration of T cells and immune checkpoint blockade enhancing cytotoxicity [106,107]. These findings also have a bearing on the potential of radiotherapy to treat PDAC. Targeted radiotherapy can drive an adaptive immune response due to the release of tumour cell antigens, however, this can be mitigated by inflammation driving stromal remodelling and a more fibrotic, immune suppressed TME. In fact, Lan and colleagues found that simultaneous targeting of TGFβ and PD-L1, in combination with radiotherapy, prolonged survival in mouse models of several cancers, including pancreatic cancer [108]. Together, these data highlight potential therapeutic avenues that could be explored to bolster TGFβ therapy.

TGFβ targeting may also have consequences in other immune cells that could be harnessed as part of combinatorial approaches. For example, TGFβ can negatively influence the tumour immune response via its capacity to inhibit the maturation and antigen-presenting ability of dendritic cells, as well as to dampen the activity of effector T cells and promote Tregs [102]. Adoptive transfer of chimeric antigen receptor (CAR)-engineered T cells is a promising novel therapeutic strategy for cancer, due to the ability to target virtually any tumour associated antigen. In PDAC, target antigens currently under investigation include integrin αvβ6, CD276, CD24, prostate stem cell antigen (PSCA), carcinoembryonic antigen-related adhesion molecule 7 (CEACAM7), MUC1, mesothelin, FAP and Her-2 [109]. However, the immune-suppressed TME has limited the efficacy of this approach in solid tumours, at least in part due to the effects of TGFβ signalling [110]. Thus, inhibiting TGFβ signalling, or even engineering TGFβ-resistant CAR T cells, could offer hope for the future of CAR T cell therapy in PDAC.

**Figure 3:**
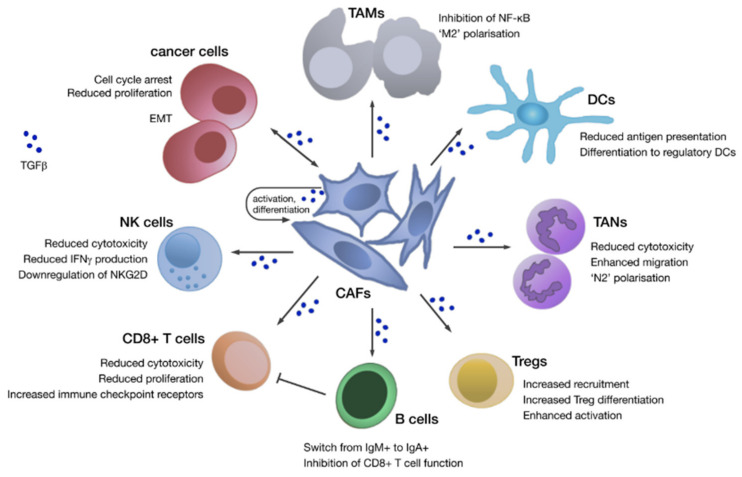
TGFβ secretion by CAFs impacts multiple cell types in pancreatic cancer.

In conducting pre-clinical and clinical trials there is now a clear need to measure the impact treatment has upon the wider TME, especially the CAFs, not only to better understand the biology, but also to reveal vulnerabilities that may suggest combinatorial or second-line strategies. For instance, the TGFβ inhibitor combinatorial trials described above have demonstrated moderate efficacy in patients, but it would of course be useful to know how therapy affected CAF subpopulations. Further study should dissect where inhibition of TGFβ signalling is having the biggest effect in these tumours, whether through disrupting CAF pro-tumorigenic function or via relieving immune suppression, or more likely, both (as illustrated in Figure 3). Further, with reciprocal signalling shown between CAFs and cancer cells [47], understanding how treatment affects this communication is key. For instance, short term SHH inhibition was shown to cause an increase in cancer cell specific pERK signalling [66], while long term gemcitabine treatment increased TGFβ signalling [97]. Understanding CAFs in the developing PDAC is essential, but so too is identifying the changes in patients who have undergone therapy, as this may expose key targets or highlight resistance mechanisms.

## 7. Concluding Remarks

Over the past few years, advances in our understanding of the heterogeneity of CAFs in the pancreatic cancer microenvironment have offered renewed hope that stromal-targeted therapies could still hold promise in this disease. It is clear from functional studies, however, that CAF heterogeneity is not restricted simply to expression of specific markers, and that populations exist that exhibit both tumour-promoting and tumour-restrictive behaviours. Therefore, rational approaches to target tumour-promoting CAF behaviour, rather than targeting all CAFs, will be required. This could be achieved by converting tumour-promoting CAFs into a more tumour-suppressive phenotype, ablation of a tumour-promoting population, targeting of specific tumour-promoting secreted chemokines or cytokines, such as TGFβ, or by a combination of these approaches. It is likely that any effective regimen will also include chemotherapy and potentially immune-targeting therapy, and that patient stratification will be important. It is clear, however, that in order to elucidate how different populations can be targeted but also to understand the effects of subtype targeting in complex systems, a great deal more work is required.
